# Imaging peripheral lymphatic dysfunction in chronic conditions

**DOI:** 10.3389/fphys.2023.1132097

**Published:** 2023-03-15

**Authors:** Eva M. Sevick-Muraca, Caroline E. Fife, John C. Rasmussen

**Affiliations:** ^1^ Brown Foundation Institute of Molecular Medicine, The University of Texas Health Science Center at Houston, Houston, TX, United States; ^2^ Department of Geriatrics, Baylor College of Medicine, Houston, TX, United States

**Keywords:** near-infrared fluorescence, lymphatic imaging, indocyanine green, rehabilitation medicine, autoimmune disease, chronic venous disease, lymphedema, lipedema

## Abstract

The lymphatics play important roles in chronic diseases/conditions that comprise the bulk of healthcare worldwide. Yet the ability to routinely image and diagnose lymphatic dysfunction, using commonly available clinical imaging modalities, has been lacking and as a result, the development of effective treatment strategies suffers. Nearly two decades ago, investigational near-infrared fluorescence lymphatic imaging and ICG lymphography were developed as routine diagnostic for clinically evaluating, quantifying, and treating lymphatic dysfunction in cancer-related and primary lymphedema, chronic venous disease, and more recently, autoimmune and neurodegenerative disorders. In this review, we provide an overview of what these non-invasive technologies have taught us about lymphatic (dys) function and anatomy in human studies and in corollary animal studies of human disease. We summarize by commenting on new impactful clinical frontiers in lymphatic science that remain to be facilitated by imaging.

## 1 Introduction

In 2010 Levick and Michel demonstrated that macromolecules, cellular waste, and capillary filtrate from the interstitium are excluded from *direct* absorption into venous blood vessels (Levick and Michel, 2010). Instead, direct recovery of fluid lost from transcapillary filtration occurs *exclusively* by peripheral lymphatics. Interstitial fluid enters the lymphatics, becoming “lymph,” and empties into the subclavian and/or jugular vein to replenish the hemovascular circulation. During transit through the peripheral lymphatics, lymph also carries antigens and antigen presenting cells to draining lymph nodes where immune activation and/or tolerance is regionally established. In these lymph nodes, a sequestered orchestration of immune cells results in finely-tuned, activated T- and B- cells as well as the generation of antigen-specific antibodies that are subsequently drained through efferent lymphatic vessels to the blood vasculature. Despite being essential to maintain tissue fluid homeostasis and systemic immunity, the peripheral lymphatic system has largely escaped modern clinical investigation due to the inability to routinely visualize or image lymph and to characterize the lymphatic vessels that carry it. Inroads to lymphatic research have been made possible by discovery of the *Prox1* lymphatic endothelial cell marker (Hong et al., 2002; Wilting et al., 2002) and the ability to perform serial sacrifice of rodent animals to explore the role of lymphatic dysfunction in animal models of human disease. However, clinical translation of preclinical research results, and clinical lymphatic research in general, are hampered by the inability to *routinely* and *non-invasively* image the peripheral lymphatics. As a result, the role peripheral lymphatics play in human health and disease is not well defined. More effective therapeutic strategies that target lymphatic dysfunction remain to be developed and deployed for prevalent chronic conditions that today largely escape successful treatment.

**FIGURE 1:**
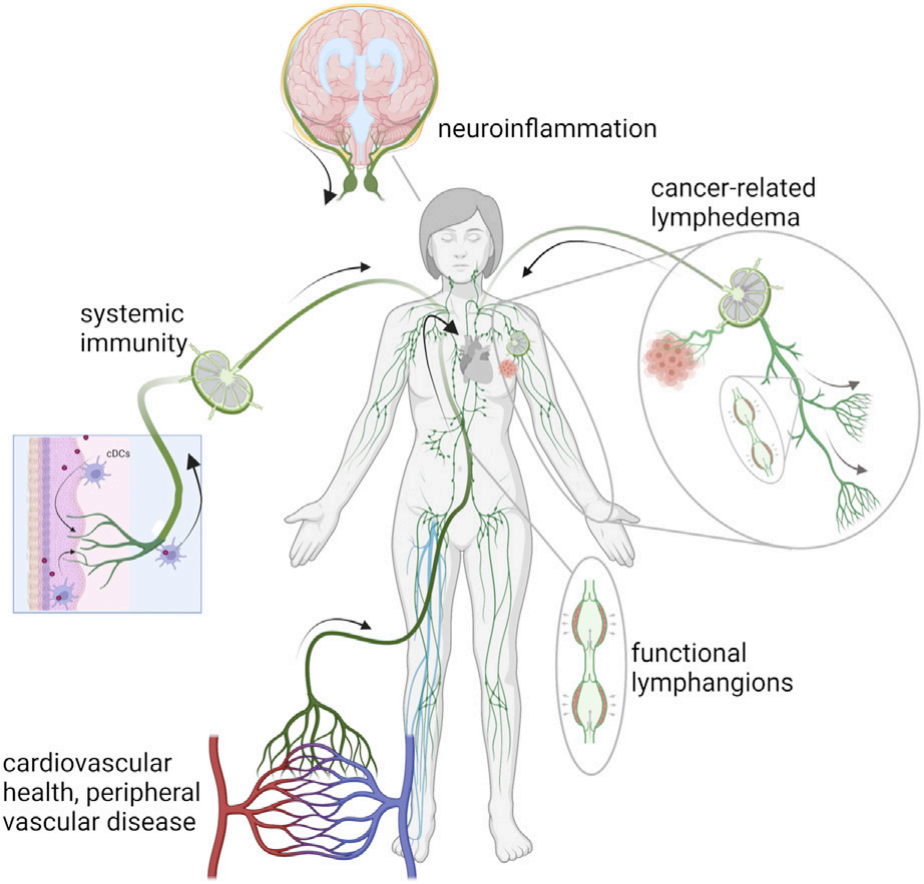
Schematic of the lymphatic vasculature in chronic conditions associated with autoimmunity, chronic venous disease, lymphedema, and neuroinflammation. Created with BioRender.

In this review we first briefly summarize conventional, clinical lymphatic imaging procedures before describing critical aspects of near-infrared fluorescence lymphatic imaging (NIRF-LI) and indocyanine green (ICG) lymphography, as well as their differences. We then focus on the clinical use of NIRF-LI/ICG lymphography for interrogating and treating chronic conditions for which lymphatic dysfunction is known or suspected. Next, we review chronic conditions for which the lymphatics are not commonly associated as part of clinical disease etiology, but which NIRF-LI/ICG lymphography suggests could provide a new, potentially a more effective target of treatment to improve outcomes. We then describe research on the role that lymphatics play in both the cause and abatement of neuroinflammation and how the lymphatics can be better harnessed to improve immunotherapies. Because of the limited tissue penetration provided by fluorescence technique, this review will not cover lymphatics in the trunk or the gastrointestinal tract that are described elsewhere (Dixon, 2010), nor do we cover the use of ICG imaging for surgical guidance. The expanse of the review is depicted in Figure 1.

## 2 Procedures for conventional imaging of the lymphatics

The visualization of the lymphatics has been challenging since the mesenteric lacteals were first described as “arteries containing milk” around 300 BC (Mayerson, 1969). Largely forgotten for 18 centuries, scientific interest in the lymphatics accelerated in the 1600s and the first known image, a colored plate illustrating the lacteals, was published by Asselli in 1627 (Aselli, 1627) (for historical perspectives on the discovery and mapping of the lymphatics see (Mayerson, 1969; Leeds, 1977; Natale et al., 2017; Suami and Shinaoka, 2019)). While the majority of these advancements were based on anatomical dissection and not a modern imaging modality, the difficulties visualizing the lymphatics remain much the same today. Both Olaus Rudbeck, in 1653, (as cited in (Mayerson, 1969)) and John Sheldon, in 1784, (as cited in (Natale et al., 2017)) noted that the challenges of studying the lymphatics included their size and fragility, lack of visual contrast rendering them nearly invisible if not filled with fluid, and the technical difficulty of cannulating them to administer exogenous contrast agents, particularly as the unidirectional flow of the lymph necessitates the administration of contrast into the smaller “branches” of the vessels. Despite great advancements in science and imaging technology in the past few decades, the lack of endogenous contrast and the challenges of introducing exogenous contrast still pose significant challenges to lymphatic imaging with conventional clinical imaging modalities, including lymphoscintigraphy, magnetic resonance, and x-ray lymphography. Below we briefly review the use of these modalities for lymphatic imaging procedures before introducing point-of-care ICG lymphography/NIRF-LI. For more details on these conventional imaging modalities, readers are referred to recent comprehensive reviews (Schwartz et al., 2020; Polomska and Proulx, 2021; van Heumen et al., 2023).

### 2.1 Lymphoscintigraphy

Sometimes used in the United States as a diagnostic for lymphatic dysfunction and more frequently so in Europe, lymphoscintigraphy is based on the collection of gamma rays emitted from the decay of radionuclide ^99m^Tc. Lymphoscintigraphy is considered the “gold standard” of lymphatic imaging. In this technique, radionuclide is indirectly administered to the lymphatics *via* intradermal or subcutaneous injection for uptake by the lymphatic plexus, a dense network of lymphatic capillaries located below the epidermis and surrounding all organs of the body. Typically, 20–45 min after injection, the subject is placed under a gamma camera and, because only one gamma photon is released for each radionuclide decay, several minutes are required to acquire a single lymphoscintigram detailing a low resolution image of the lymphatics. Sequential lymphoscintigrams are used to determine the transit time of the radionuclide from the injection sites to the draining nodal basin as a measure of lymphatic function. While lymphoscintigrams provide images of major lymphatic trunks, deep lymph nodes, and, in the case of disease, may indicate areas of blockage and/or the dermal backflow of the radionuclide, the poor spatial and temporal resolutions limit the ability to visualize smaller lymphatic vessels or active lymphatic propulsion within conducting vessels. In addition, the use of ionizing radiation poses risk to patient and clinician alike, limiting its use in routine and longitudinal studies.

### 2.2 X-ray lymphography

X-ray lymphography was perhaps the first use of commonly available clinical imaging techniques to be used for lymphatic imaging (Kinmonth, 1952). This modality is accomplished by first intradermally administering blue dye into the interdigital webbing for uptake by the lymphatic plexus. A surgeon then identifies and surgically isolates a blue-stained lymphatic vessel downstream from the injection site. The vessel is cannulated and an iodinated contrast agent is directly administered into the lymphatic vessel for x-ray imaging. While exquisite images of the lymphatics are possible using this technique, it is rarely performed today owing to the technical skill required to isolate and cannulate the lymphatic vessel. The most common modern use case is the imaging of deep lymphatics structures, such as the thoracic duct, for surgical planning. X-ray computed tomography is not routinely performed owing to the high dose of radiation required, but can be accomplished following administration of a contrast agent, either *via* cannulation of a lymphatic vessel or intranodal injection. For a more comprehensive description of x-ray lymphography see (Schwartz et al., 2020).

### 2.3 MR lymphography

When used to image lymphatics, magnetic resonance imaging (MRI) is frequently referred to as MR lymphangiography or lymphography (MRL) and may be performed with or without contrast depending on the desired image set. Non-contrast MRL is most commonly used to image the central conducting vessels or dilated lymphatic vessels based upon the high signal intensity of static or low flow lymph in heavily weighted T2 sequences that attenuate signal from background tissues. The technique typically does not resolve thin lymphatic vessels. Contrast-enhanced MRL can resolve smaller lymphatic vessels when sufficient contrast is taken up following intradermal, subcutaneous, or intranodal injection of a gadolinium (Gd)-based contrast agent. By acquiring sequential image sets, the transport of the contrast through the lymphatics over time can also be assessed. However, one of the major challenges to contrast-enhanced MRL is that low molecular weight Gd-based agents can rapidly egress into the blood vasculature causing background signals that obscure lymphatic vessels. In addition, the fate of Gd into organs, in particularly the brain, have raised concerns over the long-term safety of Gd-based contrast agents and some regulatory guidelines (such as those in Japan) encourage the use of macrocyclic over linear Gd-based agents, as less deposition is observed with these structures (Cowling and Frey, 2019; Kanda, 2019; Lancelot and Desche, 2020). For more comprehensive reviews of MRL see also (Guerrini et al., 2020; Misere et al., 2020; Calderwood et al., 2021; Forte et al., 2021; Mills et al., 2021; Salehi et al., 2022).

### 2.4 Photoacoustic

Photoacoustic imaging is an emerging imaging modality that is being translated for clinical lymphatic imaging. In this technique pulsed laser light is scanned across the surface of the skin. As the pulsed laser light travels deeply through tissues, it is absorbed by molecules (such as hemoglobin or an exogenous contrast agent) and converted to heat which causes transient thermoelastic expansions within the tissues and produces pressure waves that can be detected using an ultrasonic transducer (Wang and Hu, 2012). The detected ultrasonic waves are then reconstructed to produce images with submillimeter resolutions over several centimeters of depth (Zhao et al., 2019). Because different molecules have different absorption spectra, differing wavelengths of light can be used to target specific molecules including oxy- and deoxy-hemoglobin as well as light absorbing exogenous contrast agents, such as indocyanine green (ICG), thus enabling the visualization and identification of both lymphatic capillaries and vessels as well as blood vessels. Many photoacoustic imaging setups have limited fields of view owing to the size of the ultrasonic transducer, however efforts are under way to develop wide field photoacoustic tomography devices (Nagae et al., 2018) that can perform lymphography over large areas in both healthy and lymphedematous subjects (Oh et al., 2022). ICG dosages range from 1.0 to 2.5 mg and pulsed laser scanning times restrict acquisition times to as long as 15 min (Suzuki et al., 2020). For a more comprehensive review of photoacoustic lymphography see (Kajita et al., 2021).

The routine uses of these conventional and emerging techniques in clinical lymphatic research and in the diagnosis of lymphatic dysfunction is limited by substantial instrumentation requirements, expense, patient risk, ability to conduct longitudinal measurements to monitor treatment response, and/or the temporal and/or spatial resolution limitations of the modality. Fluorescence imaging modalities offer point-of-care approaches that do not involve ionizing radiation, offer unprecedented temporal resolution, and when compared to lymphoscintigraphy, provide improved spatial resolution for evaluating peripheral lymphatics.

## 3 Near-infrared fluorescence lymphatic imaging (NIRF-LI) or ICG lymphography

Fluorescence is a phenomenon where a molecule absorbs excitation light and, upon relaxation, reemits light of lower energy (i.e., higher wavelength). After a fluorescent molecule has relaxed to the ground state it can be repeatedly excited thereby providing greater photon counts than possible with the single emission events from decay of radionuclides as measured with lymphoscintigraphy. Lymphatic imaging of preclinical models using fluorescent contrast agents or dyes has been demonstrated over the years. For a more comprehensive review of these approaches and more particular of the various fluorescent contrast agents used preclinically, (i.e., most are not approved for human use) see (Russell et al., 2022).

As early as 1981, Bollinger *et al.* visualized the lymphatics in the feet and ankles of human subjects using florescence microscopy following subepidermal injection of FITC-labeled dextran (Bollinger et al., 1981). While these “microlymphangiography” and similar techniques have noted differences between lymphatic capillaries in healthy and lymphatic-compromised patients (Bollinger et al., 1981; Bollinger et al., 1989; Bollinger and Amann-Vesti, 2007), their clinical adoption have been limited by the physical limitations of optical imaging within the body itself. FITC or fluorescein is excited by visible light to probe the initial capillary plexus, but has limited tissue depth penetration preventing visualization of deeper conducting vessels or lymph nodes. Owing to the absorption and scattering properties of the tissues, the depth of photon penetration in the skin is wavelength dependent–ultraviolet/visible light is limited to microns of tissue depth; red light is limited to millimeters of tissue depth; while beyond the visible spectrum in the near-infrared (NIR) range (>780 nm) light penetration is the order of centimeters. NIR imaging provides opportunities for peripheral lymphatic imaging, including imaging axillary lymph nodes as deep as 3–4 cm below intact tissue surfaces (Sevick-Muraca et al., 2008). However, deep lymphatics, such as the central lymphatics, are not visualized using optical imaging techniques owing to the attenuation of light in the centimeters of tissues separating them from the skin.

**FIGURE 2:**
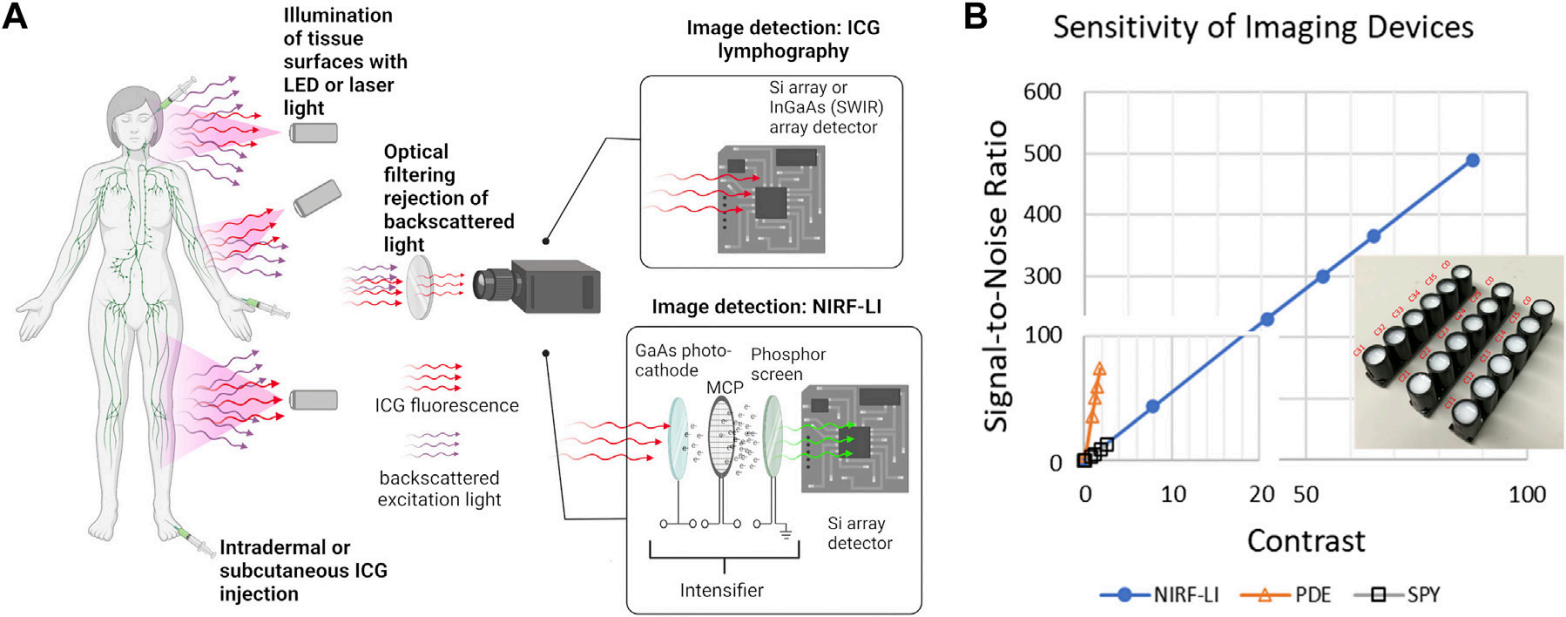
**(A)** Simplified schematic of near-infrared fluorescence imaging for non-invasive monitoring of lymphatic anatomy and function that employs ICG fluorescent contrast administration, illumination of tissues, optical filtering of backscattered excitation light, and collection of fluorescent light for imaging using ICG lymphography or near-infrared fluorescence lymphatic imaging (NIRF-LI). Created using Biorender. **(B)** Use of solid working standards (insert) for comparing performance between intensified (NIRF-LI) and commercial, non-intensified near-infrared fluorescence devices Hamamatsu PDE and Novadaq ISPY, reproduced from (Zhu et al., 2014; Aldrich et al., 2020).

Near-infrared fluorescence lymphatic imaging (NIRF-LI) and ICG lymphography are closely related imaging technologies that collect a NIR fluorescent signal emanating from the lymphatics following the off-label administration of ICG. Using this approach, the peripheral lymphatic vessels can be visualized in health and disease. As shown in Figure 2A, the instrumentation needs for ICG lymphography are fairly simple, with the minimum requirements including i) an excitation source, typically 760 nm LEDs or a diffused 785 nm laser diode beam, to illuminate the skin and excite the ICG-laden lymph; ii) optical filters to selectively pass the resultant fluorescence signal (830 nm) and reject the reflected excitation light and iii) a charge-coupled device (CCD) or scientific complementary metal-oxide semiconductor (sCMOS) silicon (Si) array detector that captures the fluorescent image. While image acquisition rates depend on configuration of the camera’s read-out electronics, image exposure times are typically measured in milliseconds, with acquisition rates ranging from 2-3 images per sec to video frame rate imaging (33 images per second) or faster. This allows imaging of non-sedated subjects, which is especially important in the pediatric population (Shibasaki et al., 2014; Tan et al., 2014; Greives et al., 2017; Pham et al., 2020). Because the instrumentation requirements are straightforward, ICG lymphography/NIRF-LI devices are compact, portable, and can be easily transported from room to room in a clinic, much like ultrasound devices. Thus, unlike the conventional and emerging imaging modalities described above, ICG lymphography/NIRF-LI devices may provide point-of-care diagnostics for routine diagnostic imaging and clinical research.

While no imaging device has been formally approved by the United States Food and Drug Administration (FDA) for the indication of ICG-based lymphatic imaging, several commercially available devices, indicated for blood perfusion imaging based on ICG-fluorescence, have been used off-label to image the peripheral lymphatics (Zhu and Sevick-Muraca, 2015; DSouza et al., 2016; Sajedi et al., 2019). In the United States, these commercially available devices include Hamamatsu’s PhotoDynamic Eye (PDE), Stryker’s SPY, and Fluoptic’s Fluobeam imaging systems. While not commercially available in the United States, Diagnostic Green’s IC-Flow Imaging System, is indicated for perfusion and lymphatic imaging in the European Union. Other lymphatic imaging systems have also been reported over the years, including the authors custom NIRF-LI system which is used with FDA clearance under the combinational IND experience.

The instrumentation required for ICG lymphography/NIRF-LI is straightforward and the wavelengths that they operate enable the same penetration depths. However component selection is crucial, as each contributes to the overall sensitivity of the system thereby affecting the dose of contrast agent required to obtain maximal image contrast with the greatest signal-to-noise ratio (SNR). For example, the GaAs image intensifier in NIRF-LI devices amplify the collected fluorescence signal and the integrating CCD or sCMOS detector zero-averages noise, resulting in an increased SNR. As shown in Figure 2B, the image intensifier improves both the contrast and SNR allowing for microdose (<100 μg) administration of ICG and visualiation of lymphatic pumping when compared to systems such as the Hamamatsu PDE Neo II and the Novadaq ISPY. In addition, optical filter performance, which deteriorates with time and can allow “leakage” of excitation light and/or ambient light contamination, also impacts device performance. Because fluorescence medical devices in general lack industry-wide performance standards, one can expect large variations in performance across different imaging devices, even those from the same manufacturer. Stable, solid phantoms (as shown in the insert to Figure 2B), comprised of TiO_2_ to mimic light scattering and quantum dots with small fluorescence cross sections in the NIR wavelengths, provide a means to assess device performance as well as to calibrate registered signals from CCD/sCMOS detectors into standardized international units that are required for all medical devices (Zhu et al., 2012; Zhu et al., 2014; Zhu et al., 2016). These phantoms serve as “working standards” and should be used to provide rigor in preclinical and clinical studies (Zhu et al., 2016; Kanniyappan et al., 2020).

The contrast agent used for ICG lymphography/NIRF-LI is ICG, a dye that has been used safely in human studies for approximately 60 years and is approved for i.v. administration, typically in boluses of 5 mg with a total dose ≤ 2 mg/kg. Clinical uses include determination of cardiac output and hepatic function as well as fluorescence angiography for surgical guidance. The fluorescent properties of ICG depend on its concentration and the solvent, with peak absorption and fluorescent emission being 780 nm and 830 nm in a dilute aqueous solution and 805 nm and 835 nm in whole blood. Because ICG is a planar molecule, at concentrations greater than 25 μM, it can “stack” together forming oligomers that quench fluorescence (Toczylowska et al., 2014). Once injected into tissues, ICG binds primarily to albumin, a protein found in large quantities in the interstitium and bloodstream (Moman et al., 2022), that may prevent oligomer formation and enhance its uptake in to the lymphatics. For clinical imaging, ICG can be intradermally (off-label) administered anywhere on the body for lymphatic uptake, but the dense network of lymphatics on the dorsal aspects of the hands and feet make these areas particularly useful and lessen the need for more painful injections in the interdigital webbing. For small animal imaging, the base of the tail and the dorsal aspects of paws are optimal sites for ICG administration (Kwon and Sevick-Muraca, 2007; Lapinski et al., 2012). For near immediate uptake of ICG into the dermal lymphatics, proper intradermal injection technique is needed in both preclinical and clinical imaging. For clinical NIRF-LI, microdose boluses of ICG (containing 25 µg per 0.1 mL saline (0.25 mg/mL ICG) in adults and 12.5 µg per 0.05 mL saline in small children) are administered in a Mantoux style injection, similar to that administered for a tuberculin screening test. For preclinical NIRF-LI, 10–50 μL of 0.25–5 mg/mL ICG saline solution is used. Deeper injections penetrating into the subcutaneous tissues deliver the contrast below the lymphatic plexus which can result in delayed uptake into the conducting lymphatics through the dermal lymphatics. Besides the discomfort associated with the needlestick and some stretching feeling in the skin, intradermal injections at these volumes (≤0.1 mL) typically engender minimal pain. When using an appropriately sensitive imaging system, larger injection volumes are generally not needed for lymphatic imaging, and may inflict unnecessary injection-related pain and/or discomfort and potentially lead to the forced entry of ICG into surrounding vasculature. Serial dilution of ICG in saline also reduces the discomfort of the injection, likely by reducing the osmolality of the ICG solution.

**FIGURE 3:**
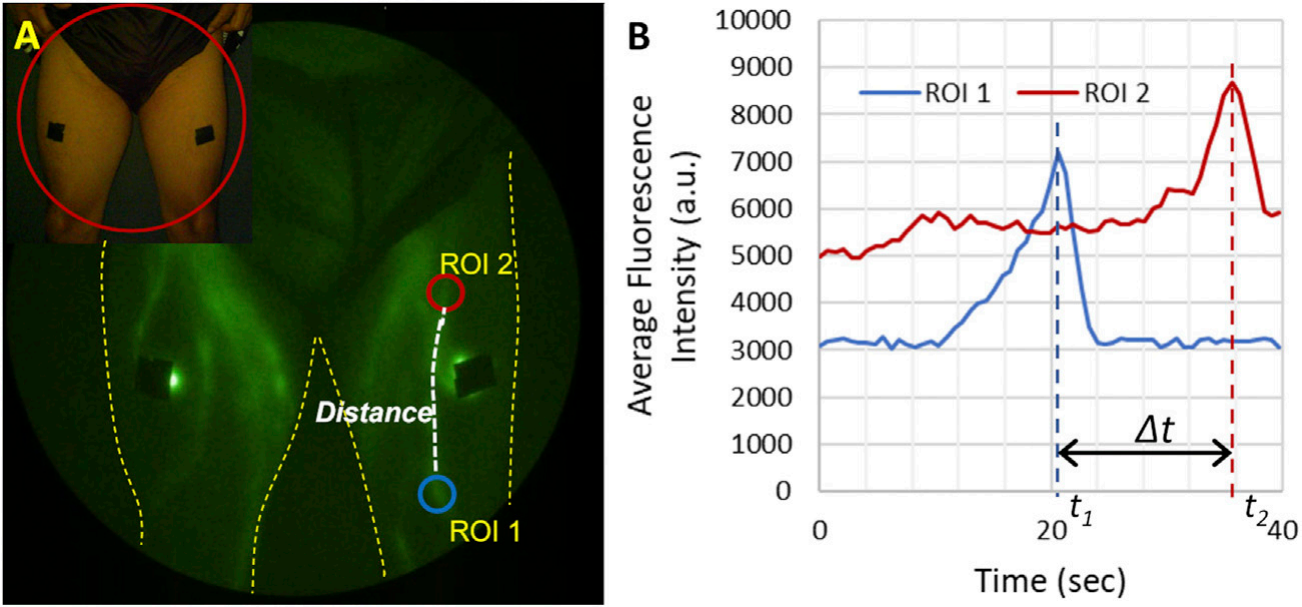
Illustration of how lymphatic propulsion is quantified. **(A)** Two regions of interest (ROIs) are selected along the lymphatic vessel and the distance along the vessel between ROIs is measured. **(B)** The average fluorescent intensity of each ROI is then plotted as a function of time. Each bolus of lymph moving through the lymphatic vessel appears as a peak in the fluorescent intensity. The velocity can be calculated as the distance between the two ROIs and the time it takes for the bolus to travel between the two ROIs (*∆t*). The propulsion rate is the total number of propulsion event observed in one ROI divided by the total imaging time.

Depending on lymphatic uptake rates, an epidermal depot of ICG can drain into the lymphatics for multiple hours, enabling ample time to visualize lymphatic function and its responses to physiotherapy or other medical intervention without the need for additional injections. The rapid image acquisition times possible with ICG lymphography/NIRF-LI enable real-time viewing of lymphatic pumping that is the hallmark of the unique physiology. ICG-laden lymph moves progressively to upstream lymphangions, or lymphatic vessel segments that are bounded by valvular structures (depicted Figure 1). These valves open and close in concert with smooth muscle contractions that are not related to heartbeat or respiration rate, but are thought to be controlled through autonomic innervation (McHale, 1990; Bachmann et al., 2019). As shown in Figure 3, Lymphatic pumping or contractile rates are computed by monitoring the fluctuation of fluorescent intensity in a region of interest (ROI) representing the influx and efflux from lymphangion segments (Sharma et al., 2007; Sharma et al., 2008; Rasmussen et al., 2009; Rasmussen et al., 2010) (Figure 3, Supplementary Video S1). While the measurement of propulsion rate allows the quantification of lymphatic function, based on the observation of propulsion events, the technique does not as yet enable the quantification of volumetric lymphatic load or transport.

While many factors likely impact pumping rate, the pressure or volume of interstitial fluids is known to directly impact lymphatic uptake and pumping. Preclinical studies show that lymphatic pumping decreases with age (Unno et al., 2011; Jakic et al., 2020) coinciding with the onset of many chronic conditions described herein. In the lower extremities, lymphatic pumping rate increases upon standing (Rasmussen et al., 2021) and is likely essential for the effective transport of lymph against gravity through the thoracic duct to the subclavian vein. By using a pressure cuff and ICG lymphography, Unno and coworkers were able to identify the lymphatic pumping pressure associated with ICG-laden transport out of the lower extremities (Unno et al., 2010), showing that reduction in pumping pressure is associated with lymphatic insufficiency. Once delivered to the blood vasculature *via* the subclavian vein, ICG is exclusively cleared by the liver (with 2–3 min normal biological half-life in blood) and secreted into the bile, providing a means to evaluate peristaltic movement in rodents and potentially in infants (Kwon and Sevick-Muraca, 2011). While there is no direct uptake of ICG by blood vessels near the injection site, over time, ICG accumulation in the blood may produce a uniform, fluorescent background signal emanating from the skin, however this background signal typically remains small and has minimal impact on lymphatic imaging. Additionally, in infants and small children, a strong fluorescent signal from the liver may be non-invasively detected. While the intradermal injection of ICG remains off label, at least in the United States, to our knowledge no severe adverse events related to intradermal injection of ICG have been reported in the thousands of patients across the world who have undergone lymphatic imaging whether for disease assessment or surgical guidance.

Recent developments of fluorescent imaging include short-wave infrared (SWIR), sometimes referred to as NIR-II imaging, in which fluorescence signals of wavelengths ranging from about 900 to 1,700 nm are collected using InGaAs scientific cameras. Significant advantages of imaging at these wavelengths are the reduced scattering of photons in the tissues and a greater, wavelength separation between excitation and fluorescent light, i.e., Stoke’s shift, that results in reduced light leakage through the optical filters. Thus, SWIR potentially allows for the acquisition sharper images of anatomic structures beneath the skin. However, an increase in water absorption at SWIR wavelengths may offset this advantage. In addition, the advantages associated with the amplification of signal and reduction of noise offered by intensified CCD or sCMOS Si detectors are not offered by SWIR systems. Perhaps the biggest hurdle however, is the lack of efficient fluorescent contrast agents approved for clinical use. While the emission spectra of ICG extends beyond 1,000 nm, the number of photons emitted in this portion of its spectra is a small fraction of that emitted at 830 nm (Starosolski et al., 2017; Carr et al., 2018; Cosco et al., 2021). Increasing the power of the incident excitation light or the dose of ICG may result in a stronger fluorescent signal from the tissues, but nonetheless is likely to be insufficient to non-invasively image deeper tissues (Zhu et al., 2020). SWIR has been deployed clinically in intraoperative studies (Teng et al., 2021; Shi et al., 2022; Wu et al., 2022) but to date, not for non-invasive clinical imaging of intact tissues.

The remainder of this review will focus upon what clinical research applications of non-invasive investigational NIRF-LI/ICG lymphography has taught us about the role of peripheral lymphatics in chronic conditions that are, and are not, traditionally associated with lymphatic dysfunction.

## 4 Breast cancer-related lymphedema

In the developed world, the chronic condition most commonly associated with lymphatic dysfunction and the first condition probed with NIRF-LI and ICG lymphography (Unno et al., 2007; Sevick-Muraca et al., 2008; Unno et al., 2008), is cancer-related lymphedema. Although the biological etiology largely remains unknown, lymphatic dysfunction is thought to occur primarily as result of lymph node dissection and/or radiation treatment. Diagnosed upon the basis of the clinical symptoms of irreversible edema (most often in limbs distal to the treatment site) and subsequent hypertrophy of tissue, cancer-related lymphedema is incurable and can proceed to fibrosis if not managed by life-long wearing of compression garments and routine treatment with manual lymphatic drainage techniques. Despite its prevalence among cancer patients and its debilitating impact, the clinical management of lymphedema has remained largely unchanged for more than 80 years with little to no medical, surgical or pharmaceutical innovation in the absence of real-time imaging to better elucidate the anatomy and pathophysiology, as well as to quantify contractile lymphatic function.

**FIGURE 4:**
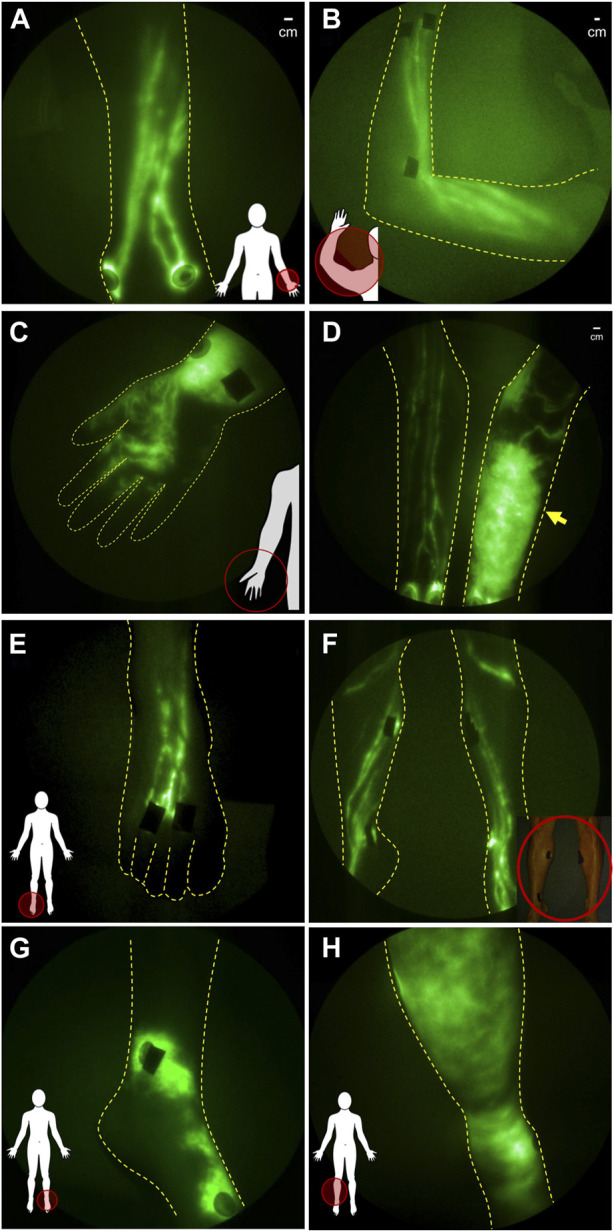
NIRF-LI studies of lymphatic drainage in **(A)** dorsal aspect of hand and **(B)** in arm of healthy normal control volunteers. **(C)** Distal flow to the palm of the hand and **(D)** dermal backflow in the forearm of affected arm compared to the normal lymphatic anatomy and function in the contralateral unaffected arm of breast cancer related lymphedema subjects. NIRFL-LI studies of lymphatic drainage in the **(E)** dorsal foot and **(F)** legs of health normal subjects and dermal backflow in **(G)** the foot and **(H)** lower extremity of patients treated with inguinal lymph node dissection and radiation treatment.

The hands and arms of breast cancer-related lymphedema subjects are characterized by the loss of straight lymphatic vessels that normally pump lymph proximally to draining axillary lymph node basins (Figures 4A, B) without drainage to the palm. In early clinical stages of cancer-related lymphedema, dilated, tortuous vessels, that appear to lack pumping function, drain backwards into the initial lymphatic capillaries within the dermis, a phenomenon termed “dermal backflow” (Figures 4C, D). Dermal backflow does not occur in healthy normal subjects. Dermal backflow generally appears distal to the irradiated, surgical sites of cancer survivors and, with progression, spreads across the entire limb including the hand and fingers. Similar lymphatic fates in the lower extremities are experienced by melanoma, prostate and other cancer survivors who undergo lymph node dissection and/or radiation (Figures 4E–H).

In early stages of both upper and lower extremity lymphedema, NIRF-LI shows that manual lymphatic drainage improves lymphatic pumping (Tan et al., 2011), consistent with the mechanism of action proposed by rehabilitation medicine specialists and the demonstrated efficacy of early-stage treatment to significantly reduce limb volumes. Because NIRF-LI/ICG lymphography identifies functional lymphatic vessels, it can also be used to direct manual lymphatic therapies to direct fluid to these functional lymphatics (Tan et al., 2011; Suami et al., 2019). In later stages of disease, manual lymphatic drainage, as well as use of pneumatic compression devices that mimic it, likewise cause proximal movement of ICG-laden lymph. However at late stages, ICG-movement appears to occur extravascularly rather than through lymphatic vessels (Adams et al., 2010)—indicative of the advanced, adverse tissue remodeling that occurs with lymphedema progression. The reason that physiotherapies may be less effective at later stages of the disease may be due to increased resistance to lymph flow in extravascular spaces as opposed to within lymph vessels. It is important to note that besides physiotherapy, there are no clinically approved approaches established to improve lymphatic pumping.

In addition, NIRF-LI has shown lymphatic dysfunction and dermal backflow in the unaffected contralateral arms of subjects diagnosed with unilateral breast cancer lymphedema (Aldrich et al., 2012), a finding that was later confirmed with ICG lymphography (Kim et al., 2022) and lymphoscintigraphy (Modi et al., 2005) and that suggests regional lymphatic dysfunction can progress to involve other lymphatic watersheds. More recently, fluorescence evaluation of lymphatic dysfunction has been expanded beyond the upper extremity to include assessment of breast tissues that also experience irresolvable edema in response to cancer treatment (Yamamoto and Yamamoto, 2022).

Emerging treatments of cancer-related lymphedema involve microsurgeries, including i) lymphovenous anastomoses (LVAs) in which lymphatics are surgically connected to veins for drainage directly into the regional venous system, and ii) vascularized lymph node transplants (VLNTs) wherein lymph nodes from a normal part of the body are excised and transplanted into the effected lymphatic watershed. While ICG lymphography is used intraoperatively to guide microvascular surgeries, there have been few studies to longitudinally follow restoration of lymphatic function (Mukenge et al., 2011). While NIRF-LI studies are currently underway to directly evaluate whether lymphatic dysfunction progresses or abates after these surgeries, initial reports indicate that LVAs and VLNTs may be curative, but are likely most effective in the same early stages that respond well to physiotherapy.

**FIGURE 5:**
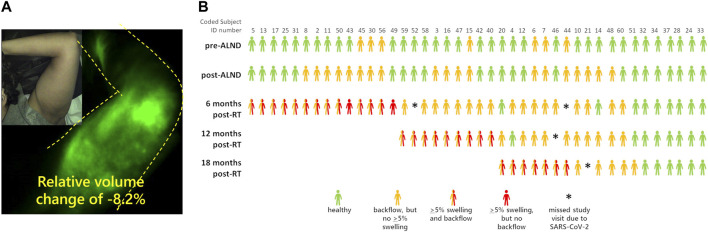
**(A)** NIRF-LI studies of dermal backflow in a breast cancer survivor who has not yet been diagnosed with lymphedema objectively based on a relative volume change of +5.0%. **(B)** Swimmer’s plot illustrating the incidence of NIRF-LI detected dermal backflow and swelling in breast cancer survivors before and as a function of time after axillary lymph node dissection and radiation therapy (RT). Adapted with permission from (Aldrich et al., 2022).

Recently, Aldrich and coworkers used NIRF-LI to longitudinally image the lymphatics of advanced breast cancer patients during their treatment journey of axillary lymph node dissection, radiation treatment, and across 6-month follow-up periods (Aldrich et al., 2022). As depicted in Figure 5, they showed that dermal backflow occurred in 83% of patients, preceding clinical symptoms of limb swelling by as much as 23 months, suggesting that the current criteria for clinically diagnosing lymphedema based on tissue volume increase is simply too late. In a mouse model of lymphadenectomy followed by radiation, Kwon and coworkers showed that persistent dermal backflow in the hindlimb was preceded by a reduction in lymphatic pumping (Kwon et al., 2019a), consistent with the clinical observations of reduced or no pumping in the arms of breast cancer survivors who later presented with dermal backflow prior to onset of clinical lymphedema symptoms (Aldrich et al., 2022). Whether physiotherapy or surgical intervention at the first sign of impaired lymphatic pumping and/or dermal backflow can ameliorate lymphatic dysfunction and whether they can prevent the irreversible tissue changes that accompany tissue volume expansion and characterize cancer-related lymphedema—remains a potentially impactful line of clinical research. It is also noteworthy that in a handful of subjects, dermal backflow preceded surgery and radiation treatment (Figure 5B), suggesting that either i) the cancer itself or neo-adjuvant chemotherapy could be causes of lymphatic dysfunction or ii) latent, subclinical dysfunction independent of cancer or cancer treatment and possibly associated with genetic predisposition, as discussed below, elevates the risk for onset of clinical symptoms after cancer treatment.

Nonetheless, these longitudinal studies support other studies that suggest ICG lymphography/NIRF-LI may provide a more accurate screening tool of cancer-related lymphedema as compared to lymphoscintigraphy (Mihara et al., 2012; Akita et al., 2013; Mihara et al., 2013; Jorgensen et al., 2021).

## 5 Head and neck cancer lymphedema

**FIGURE 6:**
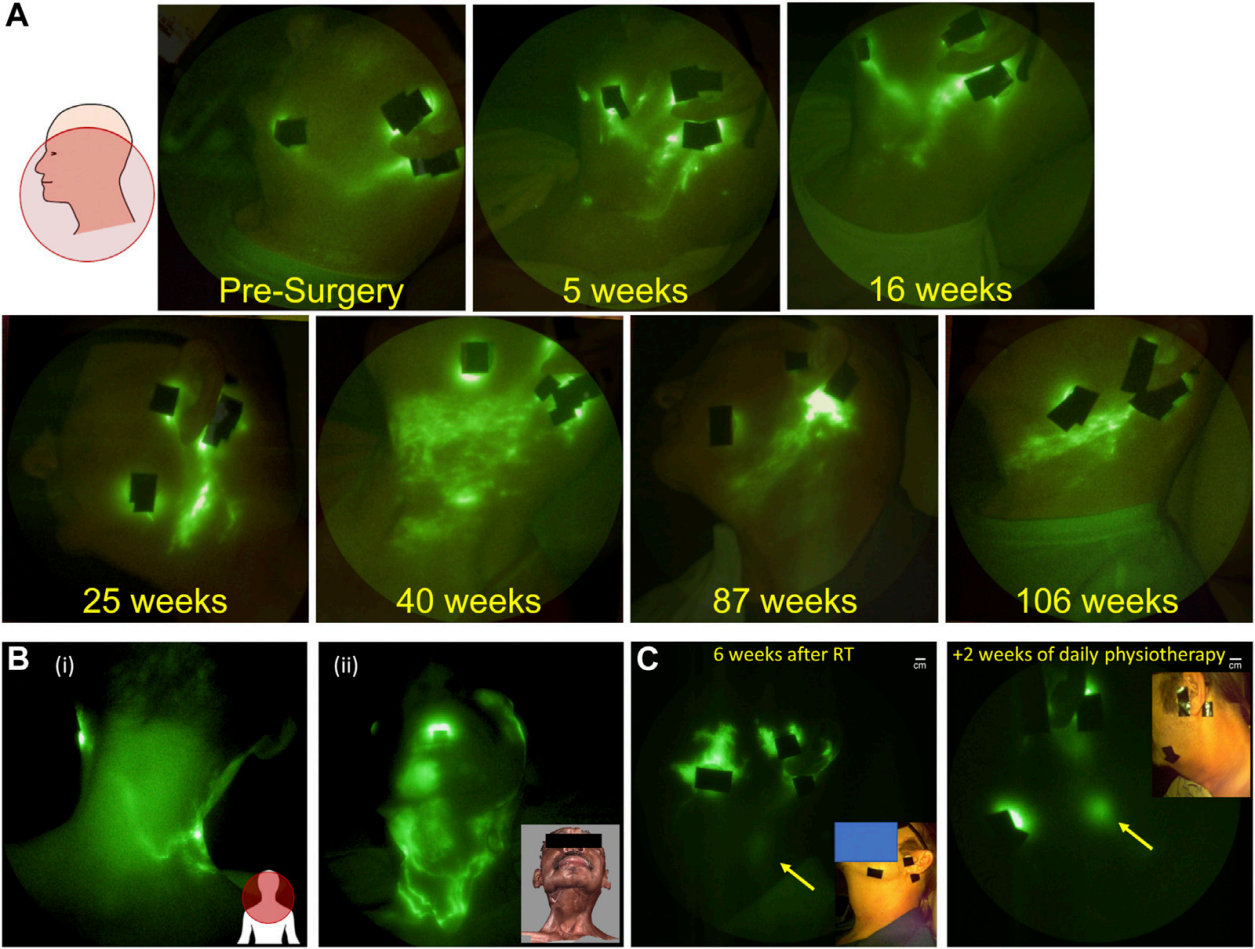
**(A)** Longitudinal NIRF-LI study showing the persistence of dermal backflow over months and years in a head and neck cancer survivor (Adapted from (Rasmussen et al., 2017); **(B)** Flow of lymph from (i) face to the back of the neck and (ii) across scar lines (Reproduced with permission from (Maus et al., 2012); and **(C)** Complete resolution of dermal backflow apparent (i) 4 weeks after completing radiation treatment and (ii) after 2 weeks of daily physiotherapy. Arrow denotes cervical lymph node that has greater enhancement after treatment (adapted with permission from (Gutierrez et al., 2019).

As described above, cancer-related lymphedema following lymph node dissection and radiation treatment for breast, prostate, melanoma, bladder, and gynecological cancers is associated with upper and/or lower extremity lymphedema which is clinically diagnosed by limb volume changes. However, head and neck cancer survivors experience lymphatic dysfunction in ways that do not always result in measurable changes in tissue volumes. Cervical lymph node dissection and radiation treatment typically cause internal lymphatic dysfunction that leads to difficulties with swallow, speech, and breathing or external lymphatic dysfunction manifesting as fibrosis, often without the obvious swelling that occurs in with upper and lower extremity lymphedema. Because microdoses of ICG do not leave permanently green-stained tissues, NIRF-LI can be used to longitudinally evaluate cranial lymphatic watersheds following ICG injections on the face and mandible. In contrast to the recent longitudinal study of breast cancer patients described above, in a longitudinal study of head and neck cancer patients, imaged before and after surgery, radiation, and during 6 months follow-ups (Rasmussen et al., 2017), ALL patients who were treated with lymph node dissection and radiation displayed developing and persistent dermal backflow in the watersheds draining the face and cervical area (Figure 6A). Dermal backflow persisted for the months and years of study duration. If dermal backflow is a harbinger of lymphedema after head and neck cancer treatment, as it appears to be in breast cancer survivors, then these NIRF-LI results are consistent with reports of 75%–90% incidence of lymphedema in head and neck cancer survivors (Deng et al., 2012; Ridner et al., 2016). These findings are of particular importance given that head and neck cancer is the fastest growing cancer type in the United States, is often diagnosed at the time of metastasis, and occurs in relatively young individuals who will potentially suffer a lifetime of morbidity after their cancer treatments.

Because objective diagnosis of head and neck lymphedema can be challenging, dermal backflow may provide the earliest indicator of lymphatic dysfunction. However, once diagnosed, the treatment of head and neck lymphedema presents additional challenges due to limited access to rehabilitation specialists offering physiotherapy for head and neck cancer survivors. Even with specialist involvement, the inability to access and evaluate internal cervical lymphatics, and the inability to identify functional lymphatic watersheds to manually direct drainage can complicate treatment. For example, therapists are traditionally trained to direct manual lymphatic drainage away from surgical scars due to a longstanding belief that lymphatic vessels cannot regrow across scar tissue. In the case of head and neck lymphedema subjects, this often means directing massage towards the back of the neck into a functional lymphatic watershed [Figure 6B (i)]. However, NIRF-LI has shown that lymphatic vessels can in fact, regrow across scar lines [Figure 6B (ii)] and consequently, manual lymphatic drainage directed towards these NIRF-LI identified functional vessels could improve drainage (Maus et al., 2012). These findings emphasize the way in which functional lymphatic imaging may help develop and deploy more efficient treatment protocols using existing therapeutic options.

In a pilot study of 10 head and neck cancer patients, we used NIRF-LI to assess the impact of early pneumatic compression intervention on lymphatic recovery about 4 weeks after completion of radiation therapy (Gutierrez et al., 2019). Eight of the 10 subjects presented with dermal backflow as indicated by NIRF-LI. Study subjects were provided with a commercially available pneumatic compression device designed to mimic manual lymphatic drainage and were asked to use it daily, at home, for 30–60 min. After 2 weeks of at home use, lymphatic dysfunction was reevaluated with NIRF-LI. In six of the eight study subjects with dermal backflow, the tissue surface areas demarked by dermal backflow appeared to decrease, with one subject experiencing complete amelioration of dermal backflow with improved drainage to cervical lymph nodes (Figure 6C). Whether restoration of lymphatic function is durable and early physiotherapy prevents adverse tissue remodeling remains to be studied in multi-center trials of larger populations. Nonetheless, if NIRF-LI/ICG lymphography can be used as an accurate measure of restored lymphatic function, then it could offer an early surrogate of outcomes and result in more efficient clinical trial design of new lymphedema treatments.

## 6 Regional inflammation/autoimmune disorders

**FIGURE 7:**
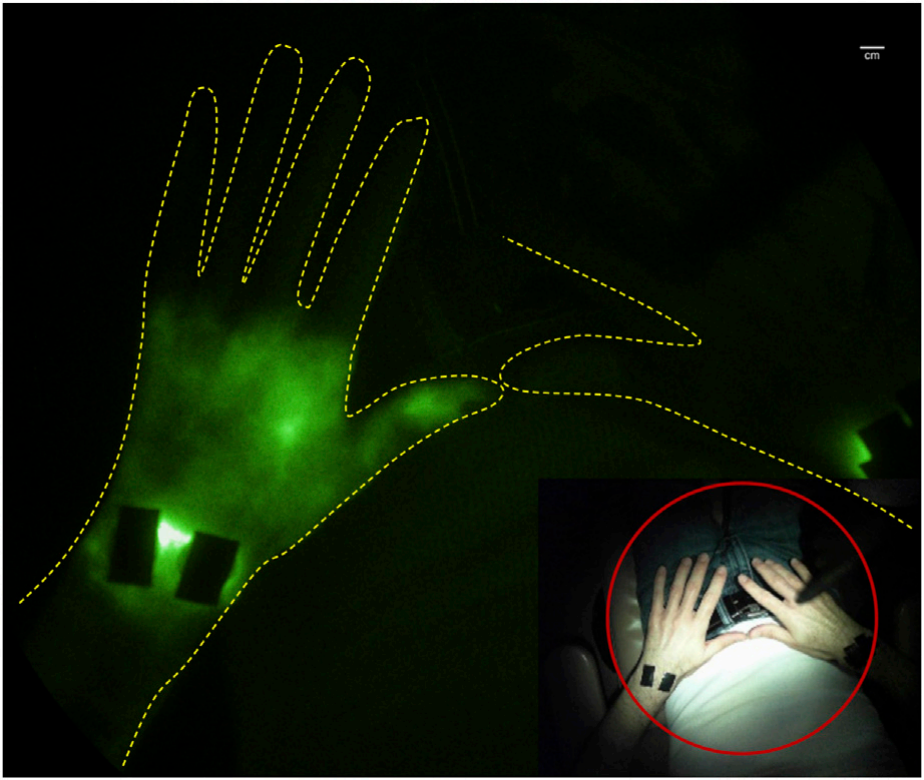
Retrograde lymph flow and dermal backflow in left hand of subject with joint pain as visualized with NIRF-LI (from study published in (Aldrich et al., 2020)).

Incidentally, one of several co-morbidities associated with rheumatoid arthritis (RA) (Joos et al., 1993) and other autoimmune disorders (Mulherin et al., 1993; Rajasekhar et al., 2013) is lymphedema. Because lymphatic vessels are essential for clearing tissue waste, transporting antigens, and mediating immune responses, it may not be surprising that the lymphatics are involved in the pathophysiology of autoimmune diseases (Schwartz et al., 2019). In transgenic and induced animal models of rheumatoid arthritis, lymphatic pumping is impaired or retrograde with paw swelling (Rahimi et al., 2016; Aldrich et al., 2017) and the draining lymph nodes exhibit expansion and subsequent collapse, possibly due to the translocation of B cells from the follicles to the sinuses, effectively clogging lymph flow (Bouta et al., 2015). An alternate or complementary mechanism may involve neutrophil migration in response to danger associated- or pathogen associated molecular patterns (known as DAMPS or PAMPs) through the afferent lymphatic vessels or through high endothelial venules into the sinuses of lymph nodes draining inflamed tissues (Hampton and Chtanova, 2016). Under inflammatory conditions, such as sterile inflammation induced by lipopolysaccharide (LPS) or long-term nitric oxide (NO) exposure (Galkina et al., 2019), neutrophils release granule proteins and chromatin to form neutrophil extracellular traps (NETs). This process, referred to as “NETosis,” that may be responsible for lymph node collapse and alteration of immune response (Kaplan and Radic, 2012) as well as contributing to impaired lymphatic drainage. Preclinical animals, dosed regionally with LPS, experience lymphatic vessel dilation, impaired lymphatic pumping, and lymphatic reflux that coincide with the rise and ebb of systemic levels of IL-1β, IL-6, and TNF-α. Indeed, subcutaneous or intraderamal introduction of IL-1β or TNF−α into the lymphatic watershed of mice results in transient impairment of pumping that is dependent upon NO (Aldrich and Sevick-Muraca, 2013). Studies of lymphatic vessels extracted from WT and TNF-transgenic mice and perfused *ex vivo* show impaired lymphatic vessel contractile pumping (Liang et al., 2021; Scallan et al., 2021), indicating that smooth muscle cell activity in lymphangions may be altered in a NO dependent fashion. Preliminary clinical imaging studies of RA flare have also demonstrated altered lymphatic function, as imaged by lymphoscintigraphy (Joos et al., 1993) and by NIRF-LI/ICG lymphography (Aldrich et al., 2020; Bell et al., 2020). Impaired drainage of ICG-laden lymph from the hands (Bell et al., 2020) with dermal backflow in the wrists and forearm (Aldrich et al., 2020) (Figure 7) suggests that draining lymphatics represent a viable target for immunotherapies to quell immune responses. Indeed lymphatic-directed treatments, including LVA (Imai et al., 2021) and manual lymphatic drainage (NCT0552937) represent emerging treatments for autoimmune disorders.

## 7 Informing genetic discovery

While the etiology of secondary lymphedema, as described in section 4 and section 5 above, is unknown, its onset follows trauma such as cancer treatment. When non-syndromic lymphedema symptoms occur, most often without apparent trauma, it is diagnosed as *primary* lymphedema. Presentation of primary lymphedema can occur at birth (*congenital*), at puberty or shortly thereafter (*praecox*), or after the age of 35 (*en tarda*). Currently, there are a handful of known gene variants associated with syndromic and non-syndromic lymphedema (Makinen et al., 2021), yet the vast majority of patients with familial, primary lymphedema do not harbor known gene variants. Historically, the disease phenotype was only identified *via* the presence of late-stage swelling with confirmation of lymphatic dysfunction by lymphoscintigraphy. Because non-syndromic primary lymphedema can have congenital, *praecox*, and *en tarda* presentation within the same family, it must be assumed that some genetic markers for primary lymphedema have not yet been identified. In a prior study, we hypothesized that we could use NIRF-LI detection of early, subclinical lymphatic dysfunction to provide a more accurate phenotype than late-stage swelling, enabling better opportunities to identify gene variants causative for familial lymphedema. Biological validation of these gene variants can also be performed by imaging lymphatic dysfunction in transgenic models that recapitulate the genetic variation.

**FIGURE 8:**
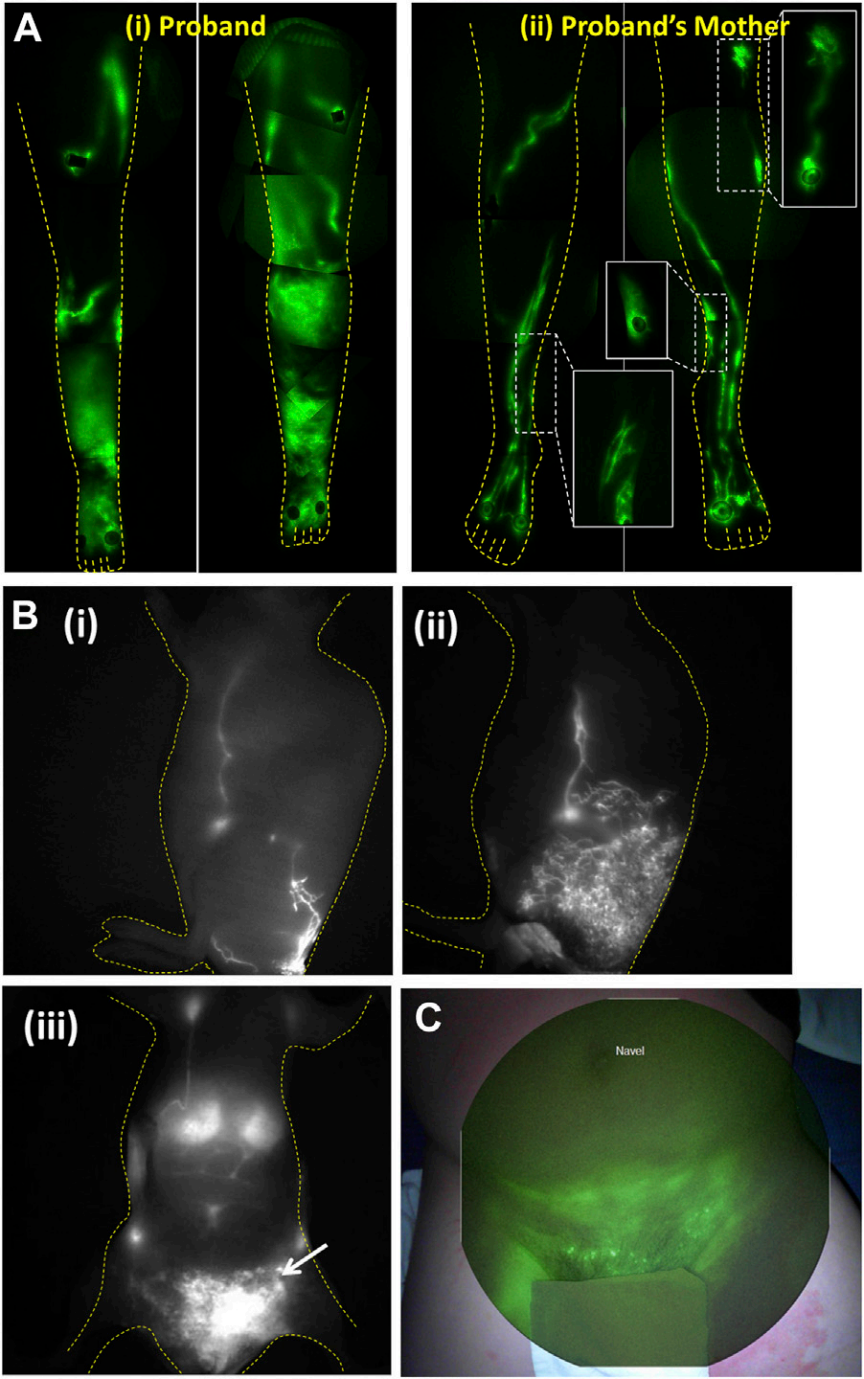
**(A)** Montages of the lower extremities of (i) proband diagnosed with primary lymphedema and (ii) her symptomatic mother demonstrating well-defined lymphatics but with dysfunctional dermal backflow expanded in figure inserts. The aberrant imaging phenotype enabled discovery of *CELSR1* as a causal gene variant associated with non-syndromic lymphedema. Reproduced with permission from (Gonzalez-Garay et al., 2016). **(B)** Lymphatic imaging of (i) lateral lymphatic draining from base of tail to inguinal and axillary lymph nodes in WT mouse, (ii) same lymphatic watershed in asymptomatic animal with *RASA1* insufficiency, that progressed with (iii) chylous ascites as seen by arrow in ventral view. Reproduced with permission from (Burrows et al., 2013). **(C)** NIRFLI overlaid on white light to show lymphatic drainage into groin and pelvic region with lymphoceles displaying bright fluorescence in a patient with RASA1 mutation. Reproduced with permission from (Burrows et al., 2013).

We used whole exome sequencing (WES) to find causative gene variants in families that harbored members with non-syndromic, congenital, *praecox*, or *en tarda* lymphedema and NIRF-LI to associate candidate genes with sub-clinical lymphatic dysfunction (Agollah et al., 2014; Gonzalez-Garay et al., 2016). Using this approach we first identified *CELSR1* as a gene variant candidate from a proband previously diagnosed with lower extremity lymphedema [Figure 8A (i)], and from her asymptomatic mother who also exhibited dermal backflow (Gonzalez-Garay et al., 2016) as shown in the inserts to Figure 8A (ii). Without NIRF-LI characteriation of lymphatic dysfunction to associate the subclinical phenotype with genotype, *CELSR1* would likely not have been attributed as a gene candidate. Most interestingly, NIRF-LI of the proband’s legs demonstrated active lymph transport through dramatic “sheet-like” waves rather than distinct vascular structures, a phenotype that remains to be explored. Today, *CELSR1* is commonly associated with lymphedema and is included in routine genetic testing (Maltese et al., 2019).

Lymphatic dysfunction also occurs in several syndromic conditions, most notably in the RAS transduction pathway (Sevick-Muraca and King, 2014; Makinen et al., 2021). Noonan (NS), cardiofaciocutaneous syndrome (CFCS), Costello syndrome (CS), capillary malformation arteriovenous malformation (CM-AVM), CLOVES (congenital lipomatous asymmetric overgrowth of the trunk with lymphatic, capillary, venous, and combined-type vascular malformations, epidermal nevi, and skeletal anomalies), Klippel–Trenaunay–Weber syndrome (KTWS), and *Proteus* syndrome (PS) all involve germline, genetic mutations in the RAS signaling pathway. In transgenic mouse models of these conditions, causative gene mutations generate lymph vessel hyperplasia and hypoplasia, chylothorax lymphangiectasia, chylous ascites, and absence of lymphatic valves. NIRF-LI/ICG lymphography can be used to characterize both mouse and human phenotypes associated with these gene mutations. For example, we demonstrated a unique phenotype of lymphatic vessel hyperplasia in a transgenic animal model of CM-AVM in which conditional knock-out of *RASA1* in lymphatic endothelial cells caused prolonged MAPK and PI3K signaling to lymphangiogenic growth factors, namely, VEGF-C (Figure 8B). The resulting proliferation of lymphatic vessels was not symptomatic until late stage onset of chylothorax in these mice, but could be alleviated through administration of anti-VEGF-C (Lapinski et al., 2012). In a subject with *RASA1* deficiency, NIRF-LI found a similar lymphatic vessel hyperplasia with disordered and impaired lymphatic transport (Figure 8C) that ultimately resulted in chylous ascites and chylothorax, and, presumably due to the absence of pharmacological interruption of lymphatic vessel proliferation, may have contributed to early death (Burrows et al., 2013).

## 8 Chronic venous disorders

Chronic venous disorders are common and are characterized by the impaired ability of veins to return blood to the heart, resulting in venous hypertension and increased capillary filtration—filtrate that must be returned to the circulation by the lymphatics. Chronic venous insufficiency (CVI) is especially impactful in the lower extremities where both venous and lymphatic return must overcome gravitational forces. CVI is descriptively classified using the clinical section of the CEAP (clinical, etiologic, anatomic, pathophysiologic) classification system. There are seven main classifications: C0, no visual evidence of venous insufficiency; C1, telangiectases (spider veins) and/or reticular veins; C2, varicose veins; C3, edema; C4, skin changes such as pigmentation (hemosiderin staining), eczema, lipodermatosclerosis, atrophieblanche, or corona phlebectatica; C5, healed venous ulcer; and C6, active ulceration. Because edema is a feature of both lymphedema and CVI, primary lymphedema can be misdiagnosed as CVI (Rasmussen et al., 2015). While the etiology for developing vascular disease is not thoroughly understood, there is increasing evidence of lymphatic involvement.

**FIGURE 9:**
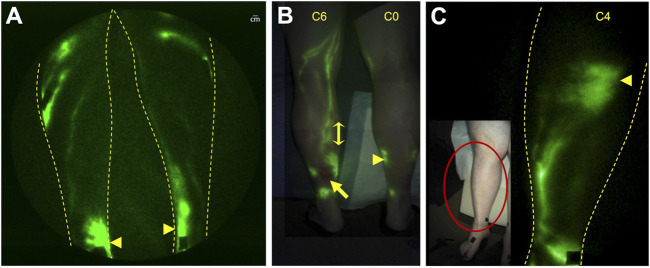
NIRF-LI in chronic venous disease. **(A)** Pooling (arrowsheads) was commonly observed in the medial ankles of subjects, including in a limb with no visible venous disease. **(B)** Lymphatic reflux (double-headed arrow) was observed in the lymphatics draining an ulcer (arrow) in the leg of one subject (See Supplemental Video S2). **(C)** Dermal backflow (arrowhead), with reduced lymphatic pumping, was observed in a subject with C4 disease. Reproduced with permission from (Rasmussen et al., 2016; Rasmussen et al., 2021).

The adventitia of blood vessels contains initial lymphatic capillaries known to be essential in the arterial wall for removal of cellular waste as well as reverse cholesterol transport by macrophages to maintain or “cleanse” it of cholesterol (Martel et al., 2013; Randolph and Miller, 2014; Kutkut et al., 2015). When lymphatic congestion occurs, intimal edema, edema in the tunica media, smooth muscle cell degeneration, and macrophage accumulation appear at developing sites of atherosclerotic plaque formation in the arteries. Likewise, leukocyte (monocyte and tissue macrophage) and lipid accumulation have also been shown within the venous wall of patients with CVI, leading to vessel wall remodeling, valvular incompetence, and subsequent venous hypertension (for review see (Nicolaides, 2005)). Tanaka and coworkers (Tanaka et al., 2012) showed that the adventitia of incompetent great saphenous veins collected from patients with CVI had abnormally high levels of lipid (presumably associated with macrophage accumulation) and fewer initial lymphatic capillaries suggesting that dysfunctional lymph transport and subsequent lymphatic congestion are associated with chronic vessel wall inflammation in CVI. In a seminal study, (Franzeck et al., 1993) used fluorescence microlymphangiography to assess the lymphatic capillary bed in the skin of patients with CVI disease. They noted more extensive dermal lymphatic capillaries in early disease, compared with that of normal subjects, which were subsequently obliterated in advanced CVI. NIRF-LI shows impaired lymphatic function pumping with abnormal lymphatic pooling and/or dermal lymphatic backflow in all limbs with C5 and C6 disease (Figure 9A), and with fewer functional conducting lymphatic vessels in subjects with the longest duration of ulceration (Rasmussen et al., 2016). In a patient with unilateral C6 disease, NIRF-LI detected actively pumping lymphatic vessels proximal to the ulcer that exhibited “reflux” (Figure 9B; Supplementary Video S2). Most surprising, dermal backflow was observed in the contralateral leg with no observable (C0) disease. NIRF-LI also demonstrated an increased incidence of dermal backflow with increased classification stage as well as reduced lymphatic pumping in C4 disease (Figure 9C), further suggesting the role of lymphatics in the etiology of CVI (Rasmussen et al., 2021).

Whether dermal backflow in C0- C4 disease portends the future development of an ulcer remains to be investigated in longitudinal studies. Indeed, the relationship between lymphatic dysfunction and the etiology of CVI is unclear. A better understanding of how inflammation, lymphatic load, high interstitial fluid pressures, fluid statis, or some combination of these impact lymphatic health and disease progression could be impactful to disease management. Associated with an aging population, venous leg ulcers are difficult to heal and frequently reoccur with substantial impact on the healthcare system (Sen et al., 2009).

Fluorescence techniques have demonstrated CVI treatments impact lymphatic function. Because lymph contains pro-inflammatory tissue waste products, their accumulation may be responsible for ulcer formation. Using ICG lymphography, Suzuki et al., demonstrated improved lymph transport from the foot to the knee after vein stripping in patients with varicose veins (Suzuki et al., 2009). Physiotherapies, such as advanced pneumatic compression, are another established approach to treat CVI. These approaches move ICG-laden lymph, which pools around ankles, towards functional lymphatic vessels and may be responsible for the accelerated healing of venous leg ulcers (Rasmussen et al., 2016). Whether physiotherapies designed to improve lymphatic drainage, can slow or prevent the progression of CVI remains to be tested, with NIRF-LI/ICG lymphography potentially providing an early surrogate endpoint to accelerate these otherwise lengthy and expensive clinical studies.

While preliminary NIRF-LI results suggests lymphatic insufficiency occurs in peripheral arterial disease (Rasmussen et al., 2021), more imaging work is needed to better understand the role of the lymphatics in the etiology of arterial disease and diabetic ulcers. Because the lymphatics are intimately associated with cardiovascular health, it may not be surprising to find that the lymphatics are part of the etiology or are impacted by other cardiovascular diseases as well (Fudim et al., 2021). For example, in patients born with a univentricular heart, the Fontan procedure redirects venous return from the lower body to the lung, often resulting in elevated central venous pressure (CVP). Elevated venous pressure can increase capillary filtration rates and possibly overwhelm the lymphatics, as well as impair the return of lymph to the hemovascular system. Upon imaging of ICG-laden lymph combined with plethysmography, Mohanakumar and coworkers (Mohanakumar et al., 2019) showed that lymphatic pumping pressure was reduced and lymphatic contractile function increased in the lower extremities of subjects with Fontan circulation when compared to controls. The clinical results are similar to the increased lymphatic pumping shown in preclinical studies of salt-induced hypertension (Kwon et al., 2012; Karlsen et al., 2018).

## 9 Lipedema and other adipose disorders

While both cancer-related and primary lymphedema result from lymphatic dysfunction, the involvement of lymphatics in the etiology of adipose disorders is not clear. There is strong evidence for the close association between lymphatics and adipose tissue as lymph nodes and lymphatic vessels are universally collocated with adipose tissues (Pond and Mattacks, 1995). In addition, lymphatic insufficiency results in subcutaneous adipose tissue (SAT) deposition in lymphedema patients as well as in the *Chy* mouse model that lacks dermal lymphatics (Karkkainen et al., 2001; Rutkowski et al., 2010), and in the *PROX-1* knock-out mouse model (Harvey et al., 2005)—evidencing the close relationship between lymphatic dysfunction and adipose tissue deposition.

Multiple common and rare adipose disorders may be associated with lymphatic dysfunction. Lipedema is a common disorder characterized by abnormal SAT accumulation that, in its initial stages, presents with easy bruising and sometimes painful, pearl-sized nodules in hypertropic subcutaneous adipose layers, while appearing exclusively in women and girls during puberty, pregnancy, or menopause. Lipedema fat deposits are generally but not always located below the waist in characteristic patterns, although the foot is spared. Lipedema can progress into lipolymphedema, a condition in which irreversible edema accompanies SAT. The disease is hereditary and resistant to weight loss. Because lymphatic dysfunction in cancer-related and primary lymphedema leads to deposition of SAT in affected limbs and in submental areas of head and neck survivors, and because the management of lipedema involves similar physiotherapies used in the treatment of lymphedema, many have postulated that lymphatic dysfunction is part of the etiology of the condition. However, when applied to early, stage I/II lipedema patients without lipolymphedema, NIRF-LI did not detect aberrant dermal backflow, but rather intact, if dilated, lymphatic vessels that “pumped” with greater frequency than a control population of similar age and BMI (Rasmussen et al., 2022). While lymphatic vessel dilation may be expected in inflammatory conditions and could precede loss of lymphatic pumping with subsequent appearance of dermal backflow, it remains unclear whether NIRF-LI/ICG lymphography would detect dermal backflow prior to progression to lipolymphedema. Nonetheless, to date, NIRF-LI does not indicate that lymphatic dysfunction is responsible for the aberrant fat deposition in the early stages of lipedema. However, some nodules found in lipedema fat appear to be fed and drained by ICG-contrasted lymphatic vessels as has been observed in some patients with another adipose disorder, Dercum’s disease (Rasmussen et al., 2014). Whether these lymphatic structures are ectopic lymph nodes, as seen in rheumatoid arthritis, and are indicative of underlying inflammatory processes remains to be studied through biopsy. Nonetheless, while lymphatic dysfunction precedes SAT deposition in primary and secondary lymphedema, these preliminary NIRF-LI studies show that in lipedema patients, lymphatic dysfunction is likely progressively caused by the inflammatory processes associated with the disease.

## 10 Chronic infectious diseases

The most common infectious cause of lymphatic dysfunction is filarial nematodes such as *Wuchereria boncrofti, Brugia malayai, and Brugia timoria.* Infecting over 200 million people across the globe, lymphedema can be triggered upon the death of adult worms in lymphatic vessels and lymph nodes. In a mouse model infected with *brugia malayi*, ICG lymphography shows lymphatic vessel dilation, lymphatic vessel proliferation, and dermal backflow (Furlong-Silva et al., 2021), consistent with clinical imaging of primary and secondary lymphedema. However other infectious diseases are also associated with lymphatic dysfunction, including *mycobacterium tuberculosis (Mtb)* and human immunodeficiency virus (HIV).

**FIGURE 10:**
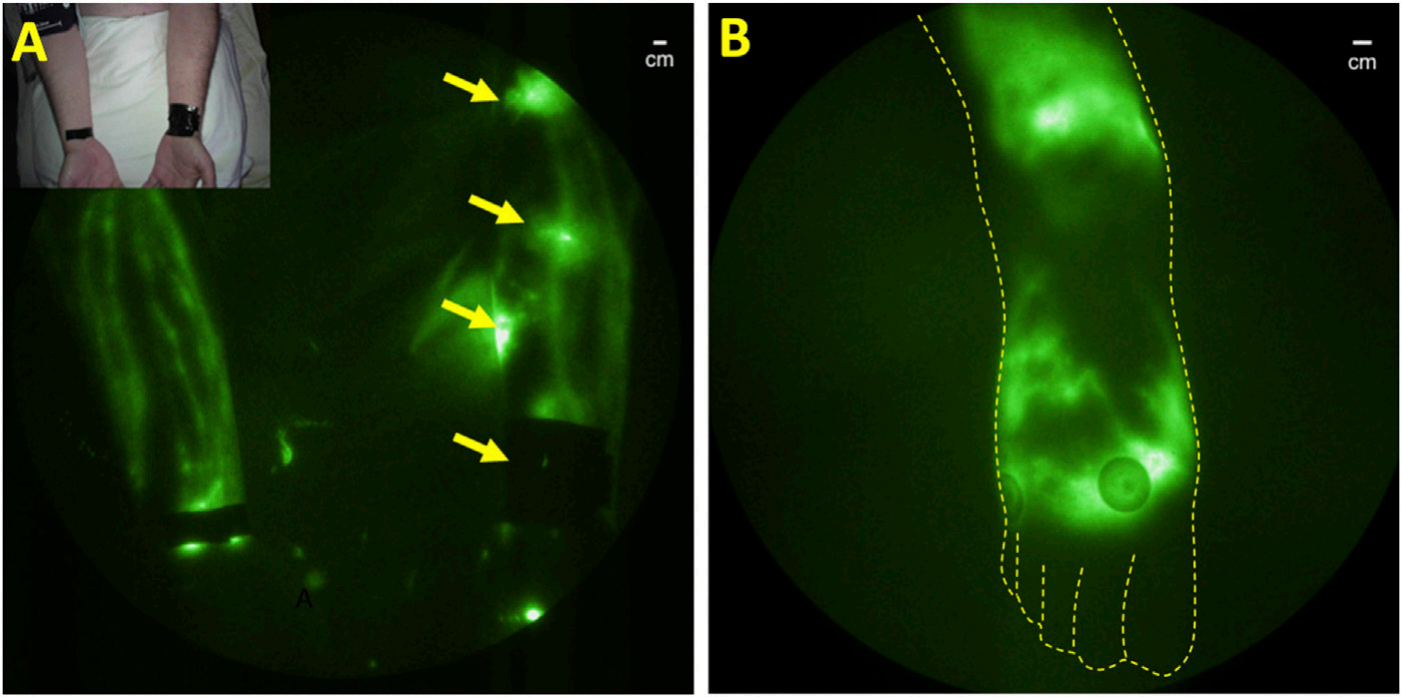
**(A)** Normal lymphatic drainage in the unaffected right arm and dermal backflow in the left arm of a subject with unresolved cutaneous *Mycobacterium tuberculosis*. Reproduced with permission from (Malek et al., 2021). **(B)** Dermal backflow in the foot of a patient with HIV with no other conditions that could be associated with lymphatic dysfunction.

Preclinical studies show that lymphatic endothelial cells themselves allow an escape from the immune system and provide a supportive niche for harboring *Mtb* infection (Lerner et al., 2016). Once outside of lymphatic endothelial cells but within lymphatic vessels, the host’s innate immune responses initiate granuloma formation through macrophage uptake of the *Mtb* and subsequent recruitment of neutrophils and other immune cells that may initially act to control infection, but ultimately forms a protective shield that induces fibrosis and immune tolerance, as well as promotes residual disease. Animal studies demonstrate lymphatic vessels serve as the earliest sites of infection with granuloma development in pulmonary lymph nodes (Basaraba et al., 2006). NIRF-LI provided the first clinical evidence of *Mtb* dissemination in an unusual clinical case of cutaneous tuberculosis (TB) following a thumb needlestick injury. After successful anti-TB medication, the subject presented with neutropenia, severe pain, and prominent lymphatic cording connecting to a fibrotic axillary lymph node later found to be PCR positive for *Mtb*. NIRF-LI was used to image lymphatics and indicated dermal backflow and dysfunctional lymphatic draining from the site of injury, and the lack of drainage through the thrombosed, cord-like structure similar to axillary web syndrome (AWS) (Figure 10A). AWS is most commonly experienced by an otherwise healthy breast cancer patients following axillary lymph node dissection and manifests impaired arm mobility. AWS has also been reported in the lymphatic watersheds draining *Staphylococcus aureus* infection (Rashtak et al., 2012). In the case of *s. aureus*, the lack of factor XIIIa staining of the cord-like structure indicates a fibrin-less thrombosis, possibly caused by NETosis, a process described above in Section 7 that can initiate coagulation in the absence of platelets. NETosis has recently been implicated in lymphatic coagulopathy in lungs and pulmonary lymph nodes as a clinical manifestation of severe COVID-19 (MacDonald et al., 2022). While NIRF-LI contributes to the first clinical evidence on the role of lymphatics in *Mtb* dissemination, strategies to direct treatments through the lymphatics to ablate the infectious agent, prevent residual disease, and preserve lymphatic function remain to be developed.

Like TB, latent HIV also resides in the lymphatics. Following initial infection, HIV spreads to regional lymph nodes within days of infection with subsequent systemic dissemination (Cohen et al., 2011; Wong and Yukl, 2016; Scholz and Kashuba, 2021). Indeed, lymphadenopathy was identified as the first early symptoms at the start of the AIDs epidemic. HIV-induced inflammatory damage to lymph node structures is attributed to the depletion of CD4^+^ T-cells and immune suppression that reduces immune responses and increases susceptibility to TB and other infections. Antiretroviral therapy can eliminate detectable viral burden in plasma, but fails to do so in lymph nodes (Kityo et al., 2018). Due to the lymphatic etiology of HIV, it may not be surprising that lymphatic dysfunction in the lower extremities occurs in HIV infection, as visualized by NIRF-LI (Figure 10B). However, it is noteworthy that lymphedema is not widely reported to be associated with HIV infection alone, although AIDs-associated Kaposi-Sarcoma, a disease of proliferative lymphatic endothelial cells, is associated with secondary lymphedema (Witte et al., 1990).

## 11 Neuroinflammation and neurodegenerative disease

**FIGURE 11:**
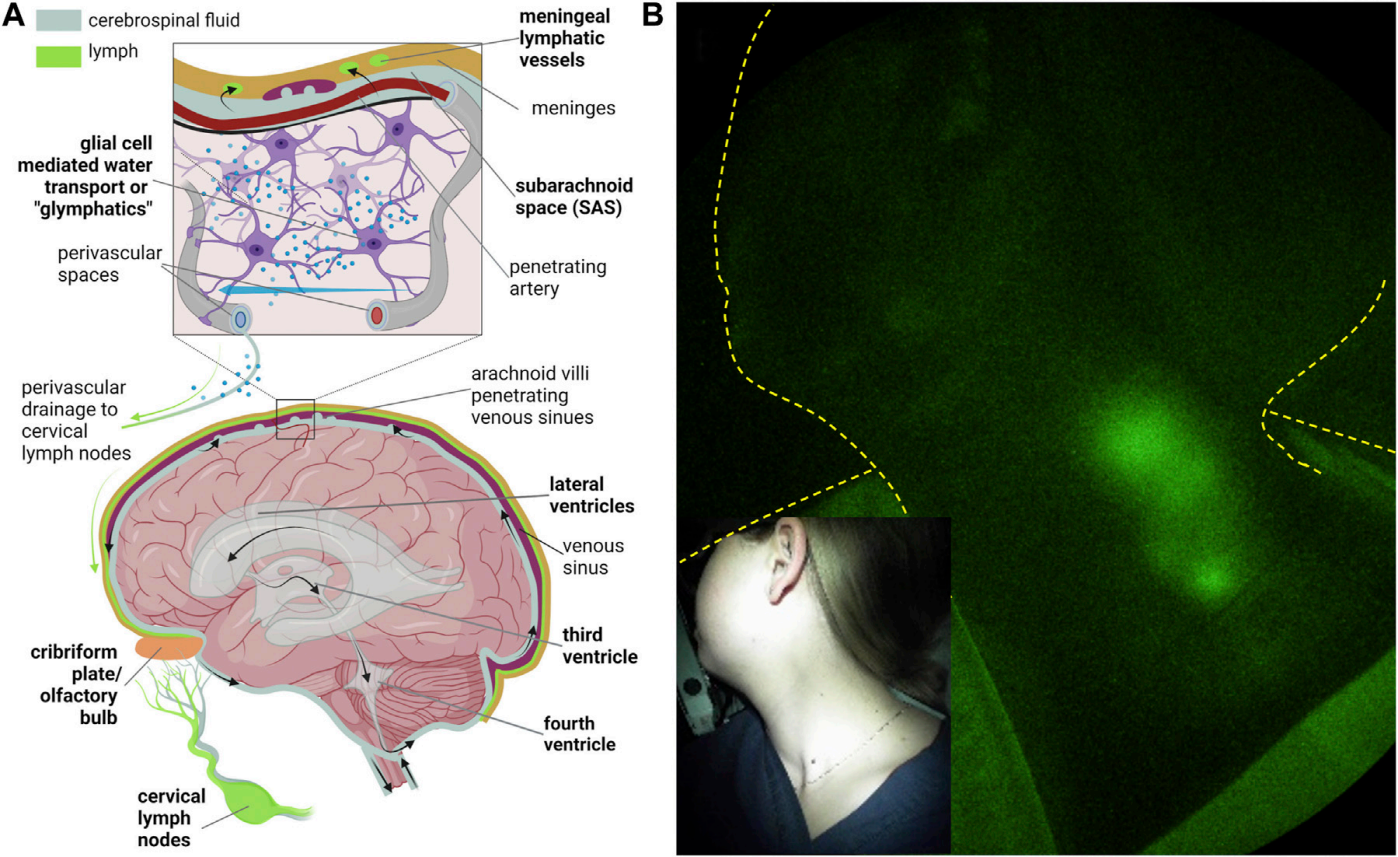
**(A)** Routes of cerebrospinal fluid (CSF) egress through the ventricular space and into the peripheral lymphatics and CSF-interstitial exchange due to glial-mediated transport (i.e., glymphatics) from arterial to venous perivascular spaces that ultimately drain into the cervical lymphatics. Created with BioRender. **(B)** NIRF-LI visualization of jugular lymph node chain following injection into palatine tonsils. Reproduced from study published in (Rasmussen et al., 2020).

Under normal conditions, cerebrospinal fluid (CSF) i) is predominantly generated in the cerebral ventricles, ii) flows from the lateral ventricles to the third and fourth ventricles, and iii) exits the fourth ventricle *via* the foramina of Luschka and Magendie into the subarachnoid space (SAS) (Figure 11A). From there, CSF has been traditionally thought to be reabsorbed by the arachnoid villi into the venous sinuses *via* Starling’s law of 1896. As discussed above, contemporary models suggest the lymphatics are responsible for the majority of interstitial fluid (ISF) or, in the case of the brain, CSF reabsorption.

The structures for absorption and drainage of CSF into the extracranial cervical lymphatics and lymph nodes include the i) lymphatic vessels near the cribriform and olfactory bulb junction (Norwood et al., 2019), ii) the meningeal lymphatics that are located parallel to the dural venous sinuses and middle meningeal arteries (Aspelund et al., 2015; Louveau et al., 2015) and at the base of the brain (Ahn et al., 2019), and iii) the perivascular (or Virchow-Robin) spaces where CSF is thought to exchange with the ISF through glial water channels (termed “glymphatics” (Iliff et al., 2012)) before emptying into basement membrane channels that drain into the cervical lymphatics (Weller et al., 2009). The anatomical and functional CSF connections between the SAS, perivascular spaces, cribriform, and the meningeal and extracranial cervical lymphatics are not well understood in humans or rodents. However, the impairment of extracranial CSF-lymphatic drainage in rodent models has been shown to contribute to the neurodegenerative changes in aging (Da Mesquita et al., 2018), Azlheimer’s disease (AD) (Da Mesquita et al., 2018; Kwon et al., 2019b), traumatic brain injury (TBI) (Bolte et al., 2020), and is postulated as a general mechanism for neurological diseases.

Rodent studies are typically performed by evaluating the lymphatic drainage of a radiolabeled tracer or fluorescent dye injected into the cisterna magna or brain parenchyma (Weller et al., 2009; Iliff et al., 2012) or by administering ICG intrathecally where pressure changes are less likely to alter CSF outflow (Kwon et al., 2017; Kwon et al., 2019b). By non-invasively monitoring the rate by which ICG appears in the cervical lymph nodes, we and others show that impaired CSF outflow is characteristic of progressive neuroinflammation (Ma et al., 2017; Kwon et al., 2019b). Longitudinal studies of transgenic AD mouse models show that peripheral lymphatic pumping function is impaired *before* the onset of amyloid deposits and reduced cervical lymph node drainage (Kwon et al., 2019b). Thus, impaired clearance of brain waste *via* the extracranial CSF-lymphatic egress may be responsible at least in part to the onset and/or progression of AD. This also suggests that neuroinflammation may have a systemic component of impaired lymphatic function that may also contribute to the etiology and/or disease progression. Anecdotally, NIRF-LI shows that persons with routine exercise schedules have improved peripheral lymphatic function, and independent reports suggest that in families with high incidence of AD, onset can be prevented or delayed with a regular exercise schedule (Meng et al., 2020).

Because ICG has never been administered directly into the CSF, the use of NIRF-LI or other fluorescent techniques to visualize CSF drainage into the peripheral lymphatics has, at writing, not been performed. The ability to image drainage into the deep jugular lymphatics has been demonstrated from ICG injections on the mandible (Figure 6C above) or in the palatine tonsils that drain directly into the jugular lymphatics (Figure 11B). In the latter, bilateral intramucosal injections of <10 μg ICG were administered in head and neck cancer subjects (Rasmussen et al., 2017) or normal subjects (Rasmussen et al., 2020) using a spinal needle. Interestingly, cervical lymph drainage in some treatment-naïve, head and neck cancer subjects appeared to be impaired, while in normal subjects, drainage was clearly evident when in the sitting as opposed to supine position. Whether afferent lymph flow to tumor-draining lymph nodes was impaired by metastasis in these patients was not evaluated. Nonetheless, in normal subjects sitting upright, gravity was found to aid cervical lymph flow. The lack flow in CSF drainage pathway in the absence of gravity, or in microgravity, may have implications for neurological health in long-term space missions.

Efforts to clinically administer ICG intrathecally or intraventricularly, using a catheter placed in standard-of-care practices following brain injury or chemotherapy instillation, must address issues of neurotoxicity. Once within the blood or lymph vasculature following i.v. or intradermal administration, ICG binds non-covalently with proteins, most notably human serum albumin. Under normal conditions CSF has considerably less plasma proteins than serum (i.e., 0.15–0.6 mg/mL in CSF versus 60–80 mg/mL in blood plasma/serum of which 50%–60% are albumins). Without protein binding and at sufficiently high concentrations, planar ICG molecules can stack together, resulting in fluorescence quenching as well as oligomer formation that, with much controversy, has been attributed to neurotoxicity (Dietz and Jaffe, 2003; Jackson, 2005). Toczylowska et al. (2014) showed that an imbalance in calcium homeostasis triggers neurotoxicity at 30 min exposures to 75–125 μM ICG in hypo-osmotic buffer media and that the mechanism of toxicity was due to ICG oligomer formation. At concentrations less than 25 μM, no toxicity was noted, suggesting that imaging systems sensitive to tissue concentrations less than 25 μM will be needed to evaluate the role of lymphatics in CSF outflow studies. Currently, our team is engaged in NIRF-LI imaging of cervical drainage and assessing ventricular dynamics with tomographic imaging adapted to fluorescence in response to ventricular administration of ICG microdoses.

## 12 Directing pharmacological delivery to the lymphatics

The immune status of tissues within a lymphatic watershed is mediated by lymphatic vessels and lymph nodes. Whether regional inflammation in the synovium of RA patients or the immune tolerance permitting cancer metastasis to regional lymph nodes, most targets of immune modulating drugs lie within the lymphatic vasculature. Except for the initial lymphatic capillaries that surround all organs, (including the epidermis) or those that are formed through the process of lymphangiogenesis, there is no other route of entry into the lymphatics. Entrance into the intact, high endothelial venules of lymph nodes is privileged for neutrophils and naive T and B cells arriving from the thymus. Because lymph empties into blood and blood does not empty into the lymphatics, conventional routes of dosing may be inadequate to maximize relevant drug targets in the lymphatics. As a result, drugs administered intravenously have a limited opportunity for maximum exposure to their targets in the peripheral lymphatics. While i.v. administered drugs extravasate through a leaky blood vasculature present in inflamed or cancer tissues, the majority of the drug is carried to liver, gut, and other tissues causing significant off-target side-effects. Subcutaneous administration below the epidermal space (also termed subcutaneous infusion (Caccialanza et al., 2018)) results in reduced drug availability associated with off-target cellular uptake and processing (Kagan, 2014) and affords limited lymph node uptake. Unfortunately, subcutaneous infusion is generally prescribed in the abdomen or thigh and are typically proximal to the affected lymphatic watershed and, therefore, do not drain to the pertinent lymph node basin.

Because lymphatic dysfunction may plague portions of the affected lymphatic watershed, we have used NIRF-LI to confirm delivery through functional afferent lymphatic vessels draining regional lymph nodes in preclinical studies as well as to monitor the response to lymphatic delivery. Using hollow microneedles to deliver drug below the epidermis, we have dosed anti-TNF-α within the lymphatic watershed draining inguinal and axillary lymph nodes of rats with collagen-induced RA in the ipsilateral hind paws that also drain to the inguinal lymph nodes. Imaging not only confirmed delivery of drug to draining lymph nodes, but also showed that lymphatic delivery significantly alleviated retrograde lymph flow and swelling in the hind paws compared to when the drug was administered systemically (Aldrich et al., 2017). By dosing drugs to *attenuate* immune responses regionally, as opposed to systemically, susceptibility to infections as well as other adverse events associated with systemic dampening immune responses may possibly be alleviated. In normal study subjects, hollow microneedles delivered ICG at volumes clinically relevant for drug administration to draining lymph nodes, suggesting feasibility for clinical translation (Kwon et al., 2019c).

Another class of drugs designed to *stimulate* the immune system involve cancer checkpoint blockade immunotherapies. Using an orthoptic tumor models that are unresponsive to systemic administration of checkpoint blockade inhibitors targeting cytotoxic T lymphocyte antigen 4 (CTLA-4) and programmed death protein 1 (PD-1), we have shown that lymphatic delivery at equivalent doses of anti-CTLA-4 or anti-PD-1 (whether through intradermal administration or microneedle devices), also results in greater anti-tumor responses including reduced or eliminated metastasis (Kwon et al., 2019c; Francis et al., 2020; Mantilla-Rojas et al., 2022). These drugs *stimulate* immune responses and, by dosing only within the tumor draining lymphatic watershed where tumor antigen is likely present, anti-tumor immune responses prevail when compared to systemic administration. With systemic administration, the immune system may be over-stimulated and dosing can result in autoimmunity and subsequent autoimmune disease. Indeed, in studies involving microneedle delivery of anti-CTLA-4 in an orthotopic mouse model of head and neck cancer, Gutkind and coworkers (Gilardi et al., 2022) showed anti-tumor responses at doses significantly lower than that needed using systemic administration. Additionally, the lower lymphatic dose reduced the lymphocyte infiltration of normal tissues that characterizes the immune related adverse events (irAEs) often suffered by cancer patients.

While systemic dosing checkpoint blockade inhibition does result in durable cancer cures, most cancer types are non-responsive and the majority of patients with cancers known to be responsive either do not benefit from these drugs, experience irAEs, and/or experience relapse. The promise of efficacious, yet reduced drug dosages that are retained within a lymphatic watershed could eliminate irAEs and expand the number of patients who benefit from current and emerging checkpoint blockade inhibitors.

Since lymph empties into blood, high lymphatic doses may lead to increased blood levels and the adverse events that accompany systemic administration. While efficacy may be maximized at lower drug doses that do not result in appreciable serum levels, it is important to note that dosing schedules are developed through pharmacokinetic analysis or plasma or serum drug levels. Thus, lymphatic targeting of drugs for autoimmune disease and cancer will require surrogate immune measures that reflect pharmacologic action with recipient lymphatic watersheds.

## 13 Conclusion and summary

In addition to their well-known importance in cancer and infection, the lymphatics play vital roles in the pathophysiology of many chronic conditions including rheumatoid arthritis and other autoimmune disorders, neurodegenerative diseases and chronic venous disease. The prevalence of these chronic conditions continues to increase and are typically managed by non-curative treatments that rarely address the role of lymphatic dysfunction in disease progression. The growing numbers of cancer survivors and the chronic conditions they face will create enormous healthcare burdens unless more effective treatment strategies are developed.

Since their identification in the 17th century, little progress had been made in the understanding of lymphatic anatomy and (dys) function until the advent of point-of-care, real-time imaging. Because chronic conditions typically do not have a clinically relevant animal model, and lymphatics in quadrapedals are not as susceptible to the same gravitational effects as bipedals (humans), it is essential that discoveries arise from clinical investigations. NIRF-LI and ICG lymphography may be used as an accurate measurement of the peripheral lymphatic anatomy and function to identify when lymphatic dysfunction plays a significant role in the etiology and progression of chronic conditions. These imaging techniques may also provide early surrogate measures of treatment outcomes, resulting in more efficient clinical trial design of new treatment strategies without the cost and infrastructure burden associated with radiological procedures or the long timeframes required for clinical outcomes. Finally, the recent recognition that (g) lymphatics serve as a “garbage truck” of the brain (Nedergaard, 2013) could be harnessed to prevent and treat acute or chronic neuroinflammation with profound societal impact. As described in this review, technological advances to image cerebrospinal fluid outflow into the peripheral lymphatics could open new opportunities to develop effective treatment strategies where few currently exist for neurological insults or neurodegenerative conditions.

It is highly likely that our understanding of lymphatic dysfunction and clinical strategies to manage it will evolve dramatically with the application of point-of-care technologies that facilitate clinical research. In addition, it is highly likely that instrumentation and drug advances will add additional capabilities than currently achievable. For example, the ability to probe tissues deeper than 3–4 cm requires new advances in optical filtering and detectors. While ICG provides a safe imaging agent, the ability to image following microdosing (i.e., <100 μg) opens up opportunities for using brighter and molecularly targeting dyes that could provide a molecular basis of lymphatic dysfunction.

In summary, the opportunities to advance lymphatic science, lymphatic imaging technologies, and the treatments of clinical conditions they affect represent the next frontier for interdisciplinary collaboration between engineers, physiologists, clinicians, and the patients they treat.

## Supplementary material

Supplementary Video S1 Normal lymphatic drainage in the lower extremities of a healthy subject.

Supplementary Video S2 Lymphatic reflux in a patient with active ulceration. Reproduced with permission from (Rasmussen et al., 2016).
